# Topical small molecule granzyme B inhibitor improves remodeling in a murine model of impaired burn wound healing

**DOI:** 10.1038/s12276-018-0095-0

**Published:** 2018-05-30

**Authors:** Yue Shen, Matthew R. Zeglinski, Christopher T. Turner, Sheetal A. Raithatha, Zhenguo Wu, Valerio Russo, Cameron Oram, Sho Hiroyasu, Layla Nabai, Hongyan Zhao, Tatjana Bozin, Kathryn Westendorf, Irina Kopko, Rachel Huang, Steve Arns, Jason Tan, Haishan Zeng, Anthony Boey, Richard Liggins, James Jaquith, Dale R. Cameron, Anthony Papp, David J. Granville

**Affiliations:** 1 Centre for Heart Lung Innovation, St. Paul’s Hospital, University of British Columbia, Vancouver, BC Canada; 2 International Collaboration On Repair Discoveries (ICORD), Vancouver Coastal Health Research Institute and Department of Pathology and Laboratory Medicine, University of British Columbia, Vancouver, BC Canada; 3 BC Professional Firefighters’ Burn and Wound Healing Group, Vancouver, BC Canada; 4 viDA Therapeutics, Inc., Vancouver, BC Canada; 5 Imaging Unit, Integrative Oncology Department, BC Cancer Agency Research Centre, Vancouver, BC Canada; 6 Photomedicine Institute, Department of Dermatology and Skin Science, University of British Columbia and Vancouver Coastal Health Research Institute, Vancouver, BC Canada; 7 Centre for Drug Research and Development, Vancouver, BC Canada

## Abstract

Granzyme B (GzmB) is a serine protease that has long been thought to function exclusively in lymphocyte-mediated apoptosis. In recent years, this paradigm has been revisited due to the recognition that GzmB accumulates in the extracellular milieu in many autoimmune and chronic inflammatory disorders, and contributes to impaired tissue remodeling due to the cleavage of extracellular matrix proteins. Knockout studies suggest that GzmB-mediated cleavage of decorin (DCN) contributes to impaired collagen fibrillogenesis and remodeling. As DCN is anti-fibrotic and contributes to reduced hypertrophic scarring, GzmB-induced DCN cleavage could play a role in wound healing following burn injury. In the present study, a novel, gel-formulated, first-in-class small-molecule inhibitor of GzmB, VTI-1002, was assessed in a murine model of impaired, diabetic burn wound healing. VTI-1002 exhibited high specificity, potency, and target selectivity. Gel-formulated VTI-1002 was able to penetrate the stratum corneum and was retained in the skin with minimal systemic absorption. Daily topical administration of VTI-1002 gel for 30 days following thermal injury showed significantly accelerated wound closure, increased DCN protein levels, and collagen organization that was translated into significantly increased wound tensile strength compared to controls. Overall, VTI-1002 gel was well-tolerated in vivo and no adverse events were observed. Topical application of VTI-1002 represents a novel therapeutic approach for the treatment of cutaneous burn wounds.

## Chronic wounds: Gel encourages healing and reduces scarring

A promising, recently developed topical treatment prevents detrimental enzyme activity in burn wounds, accelerates healing and reduces scarring. Chronic wounds that do not heal fully and require ongoing medical attention are common in the elderly, obese, and/or diabetic populations, resulting in enormous costs to healthcare services. Recent research indicates that an enzyme called granzyme. (GzmB) accumulates in chronically inflamed wounds, impairing collagen organization and disrupting tissue remodeling. David Granville at the University of British Columbia, Vancouver, Canada, and co-workers trialed a topical gel designed to inhibit GzmB activity in burn injuries in diabetic mice. When applied to the wounds for 30 days, the gel improved wound closure and remodeling while reducing scarring. The gel targeted wounds directly with no effect on surrounding tissues, and no adverse effects were observed during treatment.

## Introduction

Chronic, non-healing wounds are unable to progress through the normal, tightly regulated sequelae of overlapping stages of hemostasis, inflammation, granulation tissue formation, and remodeling. In many cases, delayed healing can be attributed to sustained inflammation and the excessive release of factors such as proteolytic enzymes that prevent re-epithelialization, de novo tissue formation, and/or wound remodeling. Chronic wounds are often associated with aging, immobility, obesity, and/or diabetes^1^. Up to 2% of the population in developed countries will experience a chronic wound during their lifetime^2^. It is estimated that treatment of chronic wounds cost US$6 to US$15 billion annually in the United States^3^. Current treatment of chronic wounds is largely underdeveloped and it still mainly comprises of conventional wound treatments with different types of advanced biomatrices and/or dressings^4^. Topical platelet-derived growth factor (PDGF) is the only biological therapeutic approved by the US Federal Drug Administration for chronic diabetic wounds. However, topical PDGF has shown limited efficacy in the clinic and its use has not been widely adopted due to the high cost and increased potential for malignancy^5^. Therefore, the production of other novel biological therapeutics for the treatment of chronic wounds is warranted. However, due to the high proteolytic environment that characterizes chronic wound beds^6–8^, success of advanced biologics has been limited.

Granzyme B (GzmB) is a member of the granzyme serine protease family. Although well known for its role in cytotoxic lymphocyte-mediated apoptosis in conjunction with the pore-forming protein perforin, in recent years, GzmB is increasingly recognized for its accumulation in the extracellular milieu in the absence of perforin, particularly in conditions associated with dysregulated inflammation and/or impaired wound healing^9–11^. Within the extracellular space, GzmB degrades critical extracellular matrix proteins that are vital for facilitating wound closure and remodeling^9,10,12,13^. Specifically, decorin (DCN) and fibronectin (FBN) have been validated as GzmB substrates in numerous in vitro and in vivo studies^9–12,14–19^. In a recent study by Parkinson et al.^12^, GzmB-generated FBN fragments induced matrix metalloproteinase-1 (MMP-1) expression in primary human fibroblasts, while GzmB-mediated DCN cleavage enhanced MMP-1-mediated and MMP-13-mediated collagen I cleavage. DCN plays an important role in collagen organization, fibrillogenesis, and tensile strength, and has been shown to be anti-fibrotic and prevent hypertrophic scarring^20,21^. Many studies have demonstrated a link between GzmB-mediated DCN cleavage and impaired collagen remodeling in a variety of disease models including skin photoaging^12^, impaired skin excisional wound healing^10,11^, and vascular injury^17,22^. Given that reduced levels of DCN and impaired collagen organization are hallmarks of hypertrophic scarring in burn injury^23^, it is plausible that inhibition of GzmB-mediated DCN proteolysis could facilitate burn wound repair and remodeling.

In contrast to MMPs and other resident extracellular proteases found in wound fluids, GzmB is one of the few extracellular serine proteases with no endogenous extracellular inhibitor currently identified in humans. This is important as extracellular proteolytic activity is tightly regulated^24^. Inhibition of GzmB using serpin A3N (SA3N), an endogenous murine protease inhibitor, has been observed in a mouse model of diabetic wound healing with favorable outcomes^10^. However, SA3N has poor target selectivity and would be predicted to be immunogenic in humans^10^. Additionally, there is no known human equivalent of murine SA3N. Thus, it is essential to develop a synthetic inhibitor that has increased specificity for GzmB and a low risk of immunogenicity in humans. The present study details the development, characterization, and evaluation of therapeutic efficacy of a novel, first-in-class, highly potent small-molecule inhibitor of GzmB (VTI-1002). The compound is formulated for topical application in a murine model of diabetic burn wound healing. The topical VTI-1002 formulation is efficacious and well tolerated with repeat administration. It significantly accelerates wound closure, increases level of DCN and collagen organization, and enhances wound tensile strength.

## Materials and methods

### GzmB enzymatic assay and *K*_i_ determination

VTI-1002 was a small-molecule inhibitor (MW ~560) generously provided by viDA Therapeutics, Inc. (Vancouver, BC, Canada). The GzmB inhibition assay was carried out as follows: Assay buffer (AB), consisting of 50 mM HEPES, pH 7.5, 0.2% (w/v) 3-[(3-cholamidopropyl)dimethylammonio]-1-propanesulfonic, and 5 mM dithiothreitol was prepared. 2× GzmB (Emerald BioSystems, Bainbridge Island, WA, USA) mix (10 nM enzyme final assay concentration) was made up for incubation with the enzyme inhibitor. GzmB was screened in a full inhibitor dose–response assay (typically 12 points, to identify the half-maximal inhibitory concentration (IC_50_) in duplicate, triplicate, or higher replicates as needed (*K*_i_ values were calculated from this inhibition data as well). Inhibitors were prepared in concentrated stock in a dilution plate and transferred to the reaction plate (black 384-well medium binding plate, Greiner Bio-One FLUOTRAC™) to allow for the desired final dose–response concentrations in enzyme inhibition assay. After mixing, the plate was incubated for a total of 30 min as follows: 5 min on the shaker at 300 RPM, 20 min covered on the bench, and finally an additional 5 min, with warming to 30 °C. GzmB substrate Ac-IEPD-AMC (California Peptide Research Inc., Napa, CA, USA) was prepared in AB at 2× the final desired concentration of 50 μM. Substrate mix was added to each appropriate well on the reaction plate, and the plate was read immediately in the TECAN plate reader (TECAN INFINITE^®^ M1000 Pro), at EX/EM 380 nm/460 nm. Assay temperature was 30 °C. Background control wells consisted of 1× AB and 50 μM substrate. Positive control wells consisted of GzmB enzyme and substrate. Data were archived and analyzed using the CDD Vault from Collaborative Drug Discovery (Burlingame, CA, USA; www.collaborativedrug.com). Where necessary, *K*_i_ values were generated by fitting to the Morrison equation using Python (software version 2.7; available at http://www.python.org) and NumPy as per guidelines defined elsewhere^25–29^.

### DCN/FBN cleavage assay

Reactions were prepared in 50 mM Tris base, pH 7.5. GzmB (200 nM; Emerald BioSystems) was incubated with VTI-1002 (50 µM, generous gift from viDA Therapeutics, Inc.) for 30 min at room temperature prior to the addition of either FBN (1.0 µg; Sigma-Aldrich, St. Louis, MO, USA) or DCN (1.0 µg; R&D Systems, Minneapolis, MN, USA) substrate. Reactions were incubated overnight at 37 °C in a water bath. The next day, 6× Laemmli loading buffer was added to each sample and the samples denatured at 95 °C for 5 min in a heat block. Solutions were collected by centrifugation and the samples separated on a 10% polyacrylamide gel under denaturing conditions. Gels were washed 2 × 5 min each in distilled water and stained at room temperature for 60 min in Coomassie Brilliant Blue R (Sigma) with gentle shaking. Destaining was accomplished using a solution of 45% (v/v) methanol and 10% (v/v) glacial acetic acid. Destaining solution was replaced every 30 min until proteins were easily visible. Images were collected using the Licor Odyssey Scanner under the 700 nm channel and processed using Image Studio (Licor Biotechnology, Lincoln, NE, USA).

### Bioanalysis of VTI-1002 in mouse plasma and skin

VTI-1002 was extracted from mouse plasma via protein precipitation technique. Briefly, proteins from 50 μL of mouse plasma were precipitated using 1% (v/v) formic acid (FA) in acetonitrile (ACN), followed by centrifugation and evaporation of 100 μL of the resulting supernatant. The sample residue was reconstituted into 100 μL of mobile phase prior to ultra-performance liquid chromatography tandem mass spectrometry (UPLC-MS/MS) analysis. VTI-1002 was extracted from skin tissue in two homogenization steps. Skin samples ranging from 25 to 80 mg were first homogenized into 100 μL of PBS, followed by extraction of the aqueous homogenate using 300 μL of 1% (v/v) FA in ACN (second homogenization step). The samples were then centrifuged, and 200 μL supernatant was removed and evaporated. The samples were reconstituted into 100 μL of mobile phase prior to dilution (if required) and analysis.

Quantification of VTI-1002 from mouse plasma and skin was performed using UPLC-MS/MS Technology (Waters Acquity Xevo TQD). Briefly, VTI-1002 was isolated on a 2.1 × 50 mm^2^ C18 column (1.7 μm) using a  gradient mobile phase comprising 10 mM ammonium acetate, pH 8.7 (adjusted with ammonium hydroxide), and ACN. Detection was performed in positive ionization mode using multiple reaction monitoring.

### Mice

Genetically diabetic male mice (C57BLKS *db/db*; stock number 000642) were obtained from Jackson Laboratories (Bar Harbor, ME, USA). All mice were housed at the Genetic Engineered Models facility at St. Paul’s Hospital, University of British Columbia. All procedures were performed in accordance with the guidelines for animal experimentation approved by the Animal Experimentation Committee of the University of British Columbia. Two days prior to burn injury, all mice were fasted for 4 h and fasting blood glucose levels of the mice were determined with OneTouch Ultra Blood Glucose Meter (LifeScan, New Brunswick, NJ, USA). Only mice with fasting blood glucose levels >200 mg/dL were considered diabetic and used in the study.

### Burn wound model

Burn wounds were induced as previously described^30^. In brief, mice under anesthesia were given a burn wound induced by vertically positioning a metal rod (25 g, 1 cm in diameter) heated to 95–100 °C by submersion in boiling water for 6 s without additional pressure on the back skin that had also been depilated 3 days before wounding. VTI-1002 used for in vivo topical application was formulated in a gel consisting of carbopol, propylene glycol, methyl paraben, and propyl paraben in acetate buffer. Vehicle control gel was using the same composition without VTI-1002. Topical application of gel or gel + VTI-1002 commenced immediately after thermal injury. VTI-1002 gel (3.6 mg/mL, 50 µL) or vehicle control gel were topically applied on top of the burn wound daily for 30 days. Digital pictures of the wound area were captured, with ruler placed below, for planimetry measurements every 3 days until the end of the experiment.

### Histology and immunohistochemistry

Five-micron sections were deparaffinized and rehydrated in the following order for histological and immunohistochemical staining: three washes of xylene, 100% ethanol, 90% ethanol, 70% ethanol, and two washes of Tris-buffered vehicle. Slides were stained with hematoxylin and eosin for evaluation of morphology and picrosirius red to detect fibrillar collagen. Immunohistochemistry was performed using rat anti-mouse CD45 antibody (BD Biosciences), rabbit polyclonal CD68 antibody (Abcam), rabbit anti-mouse vimentin antibody (Cell Signaling), rabbit anti-mouse α-smooth muscle actin antibody (Abcam), anti-pan keratin antibody (Abcam), rabbit anti-collagen I antibody (Abcam), rabbit anti-collagen III antibody (Abcam), rabbit anti-FBN antibody (Abcam), and goat anti-mouse DCN antibody (R&D Systems, Minneapolis, MN, USA) as described previously^10^. Histological evaluation was performed independently by two experimental pathologists who were blinded to the experimental conditions.

### Second-harmonic generation microscopy and collagen analysis

Collagen in the tissue was visualized using a customized video rate multimodality multiphoton microscopy system^31^. Image acquisition was carried out at 15 frames/s with a resolution of 512 by 512 pixels. The laser source was an 80 MHz Ti:Sappire femtosecond laser (Chameleon, Coherent Inc., Santa Clara, CA, USA) with a wavelength tuning range of 700–950 nm. The fast imaging speed was realized by using an 8 kHz resonance scanner for the fast axis and a galvanometer scanner for the slow axis. A ×60 (NA = 1.0) water-immersion objective (LUMPLFLN60X/W, Olympus Canada, Markham, ON, Canada) was used to focus the laser light on the sample. The second-harmonic signal was collected in the epi-direction by the same objective and was then reflected by a dichroic mirror (FF665-Di02-25 × 36, Semrock, Inc., Rochester, NY, USA) and focused into a photomultiplier tube (PMTs, H9433MOD-03, Hamamatsu Corp., Bridgewater, NJ, USA). A band-pass filter (FF01-390/40-25, Semrock, Inc.) was located in front of the PMT with a transmission range from 370 to 410 nm for second-harmonic generation (SHG) detection with an excitation wavelength of 800 nm. Acquired images were averaged every 10 frames to improve the signal-to-noise ratio. The total SHG signal intensity values were quantified by ImageJ 1.5i.

### Tensile strength measurement

Strips of skin were assessed using tensiometry to determine skin strength using the Mecmesin Advanced Force Gauge AFG-100N (Mecmesin, Slinfold, UK) with a motorized test stand (Mecmesin M1000E, Mecmesin) at a speed of 3 cm/min following the manufacturer’s instructions.

### Statistical analyses

Quantitative values presented in bar graph are expressed as mean ± SEM. Quantitative values presented in box-and-whiskers plot are expressed as median and min/max value. Statistical analysis was performed using GraphPad Prism version 5.01 (GraphPad Software, San Diego, CA, USA). Wound healing curves were assessed using two-way analysis of variance (ANOVA) with Sidak’s multiple comparison test. Wound classifications were assessed by *χ*^2^ test. For all other experiments, unpaired Student’s *t* test was used for statistical analyses. For all tests, *P* < 0.05 was considered significantly different.

### Data availability

All data generated or analyzed during this study are included in this published article (and its Supplementary Information files).

## Results

### In vitro activity of VTI-1002

As shown in Table 1, VTI-1002 is a highly selective and potent inhibitor of human GzmB (*K*_i_ ~ 4.4 nM). VTI-1002 is nearly 20 times more potent against human GzmB than Ac-IEPD-CHO (*K*_i_ = 80 nM), the only commercially available covalent reversible GzmB inhibitor. GzmB preferentially cleaves proteins following aspartic acid residues, and shares a high degree of substrate similarity with caspase-8, where the optimal tetrapeptide (P4–P1) sequence is IEPD^32^. A critical challenge for teasing out the individual activities of GzmB and caspase-8 is the lack of specificity in available inhibitors. VTI-1002 was designed to maintain significant specificity for human GzmB with minimal activity against other proteases, including caspase-8. To assess specificity for human GzmB, VTI-1002 was tested against numerous relevant proteases, including caspases 3–10, cathepsin G, and neutrophil elastase (Table 1). VTI-1002 exhibited strong selectivity for GzmB. Against all enzymes screened, including caspase-8, VTI-1002 exhibited minimal inhibition at concentrations ≤300 μM in vitro (Table 1). A pathological role for GzmB in conditions associated with aging, chronic inflammation, and impaired tissue repair due to extracellular matrix (ECM) cleavage is well documented^11^. To test whether VTI-1002 effectively inhibits ECM cleavage by GzmB, in vitro cleavage assays for two key ECM proteins, DCN and FBN, were performed. As shown in Supplementary Figure 1, GzmB cleaved recombinant human DCN and human FBN, which were inhibited by pre-incubation with VTI-1002.

**Table 1:** Biofunctional characteristics of VTI-1002

**GzmB inhibition potency**			
Human GzmB (*K*_i_):	4.4 ± 2.0 nM	Mouse GzmB (IC_50_):	179 ± 18 nM

**Target selectivity**			
**Protease**	**IC**_**50**_	**Protease**	**IC**_**50**_
Caspase-3:	No inhibition	Caspase-9:	»300 μM
Caspase-4:	No inhibition	Caspase-10:	No inhibition
Caspase-5:	No inhibition	Cathepsin G:	No inhibition
Caspase-7:	No inhibition	Neutrophil elastase:	No inhibition
Caspase-8:	»300 μM		

### Topical application of VTI-1002 gel accelerates diabetic burn wound closure

**Fig. 1:**
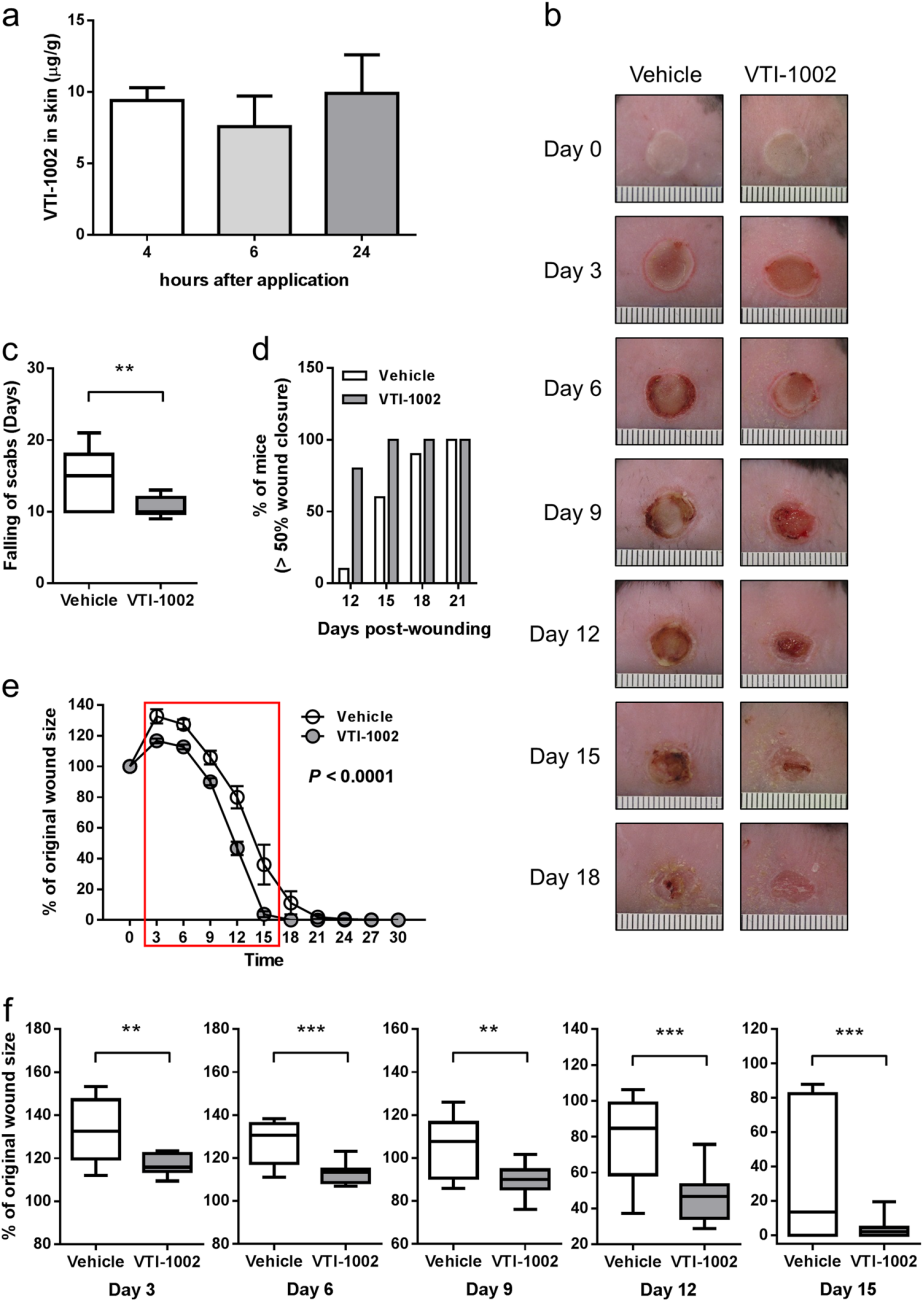
Topical application of VTI-1002 gel accelerates diabetic burn wound closure. **a** VTI-1002 is retained in diabetic skin after single topical application. **b** Representative digital images of diabetic wounds treated with vehicle or VTI-1002 gel captured over first 18 days wound healing period. **c** Comparison of scab falling-off days between vehicle control group and VTI-1002 treatment group (*n* = 10 per group). Results presented in bar graph are expressed as mean ± SEM. Results presented in box-and-whiskers plot are expressed as median and min/max value, **P < 0.01 by Student's *t* test. **d** Percentage of mice achieving over 50% wound closure in vehicle control and VTI-1002 treatment group. **e** Comparison of wound size over the 30-day healing period between vehicle control group and VTI-1002 treatment group (*n* = 10 per group). Results are expressed as mean ± SEM, *P* < 0.0001 by two-way ANOVA. Red rectangle indicates the window when significant differences at each day are observed between vehicle control group and VTI-1002 treatment group. **f** Comparison of wound size at day 3, day 6, day 9, day 12, day 15, and day 18 between vehicle control group and VTI-1002 treatment group (*n* = 10 per group). Results are expressed as median and min/max value, *P < 0.05, **P < 0.01, ***P < 0.001 by Student's *t* test

Prior to clinical trial in humans, it is essential to establish the efficacy and safety profile of VTI-1002 in animal models. Despite potential species-specific functional and structural differences between human GzmB and mouse GzmB^33^, VTI-1002 exhibits potent inhibition against mouse GzmB (IC_50_ = 179 nM, Table 1), thus facilitating target validation and appropriate efficacious evaluation in relevant mouse models. To determine whether VTI-1002 could be delivered directly to the skin in a gel formulation and be retained at the application site for a sufficient period of time, VTI-1002 was prepared in a gel formulation and applied directly to the skin. VTI-1002 was detected in the skin 4, 6, and 24 h after a single application of the gel, and there was no significant change in VTI-1002 concentration in the skin from 4 h to 24 h post-application (Fig. 1a). Plasma level of VTI-1002 was at or below the level of quantitation at any time point (Supplementary Table 1).

Based on these data, a daily application treatment regimen was implemented to investigate the therapeutic efficacy of VTI-1002 gel on diabetic burn wound healing. Animals treated with topical VTI-1002 gel exhibited faster wound closure compared with animals treated with vehicle control (Fig. 1b). The average time to the falling of scabs, an important marker of wound closure, in the VTI-1002-treated group was approximately 4.5 days earlier than in the vehicle controls (Fig. 1c). VTI-1002-treated mice also reached the 50% wound closure end-point faster than vehicle controls (Fig. 1d). Quantification of the wound area at different time-points revealed that wound area initially expanded in the first 3 days and then gradually decreased in both groups. VTI-1002-treated mice exhibited significantly improved wound closure compared to vehicle controls over 30 days (Fig. 1e, *P* < 0.0001 by two-way ANOVA). A significant difference in wound size between the two treatment groups appeared at day 3 and continued until day 15 (Fig. 1f). By day 30, the majority of mice in both treatment groups had achieved full wound closure.

### Topical application of VTI-1002 gel accelerates re-epithelialization and wound maturation in diabetic burn wounds

**Fig. 2:**
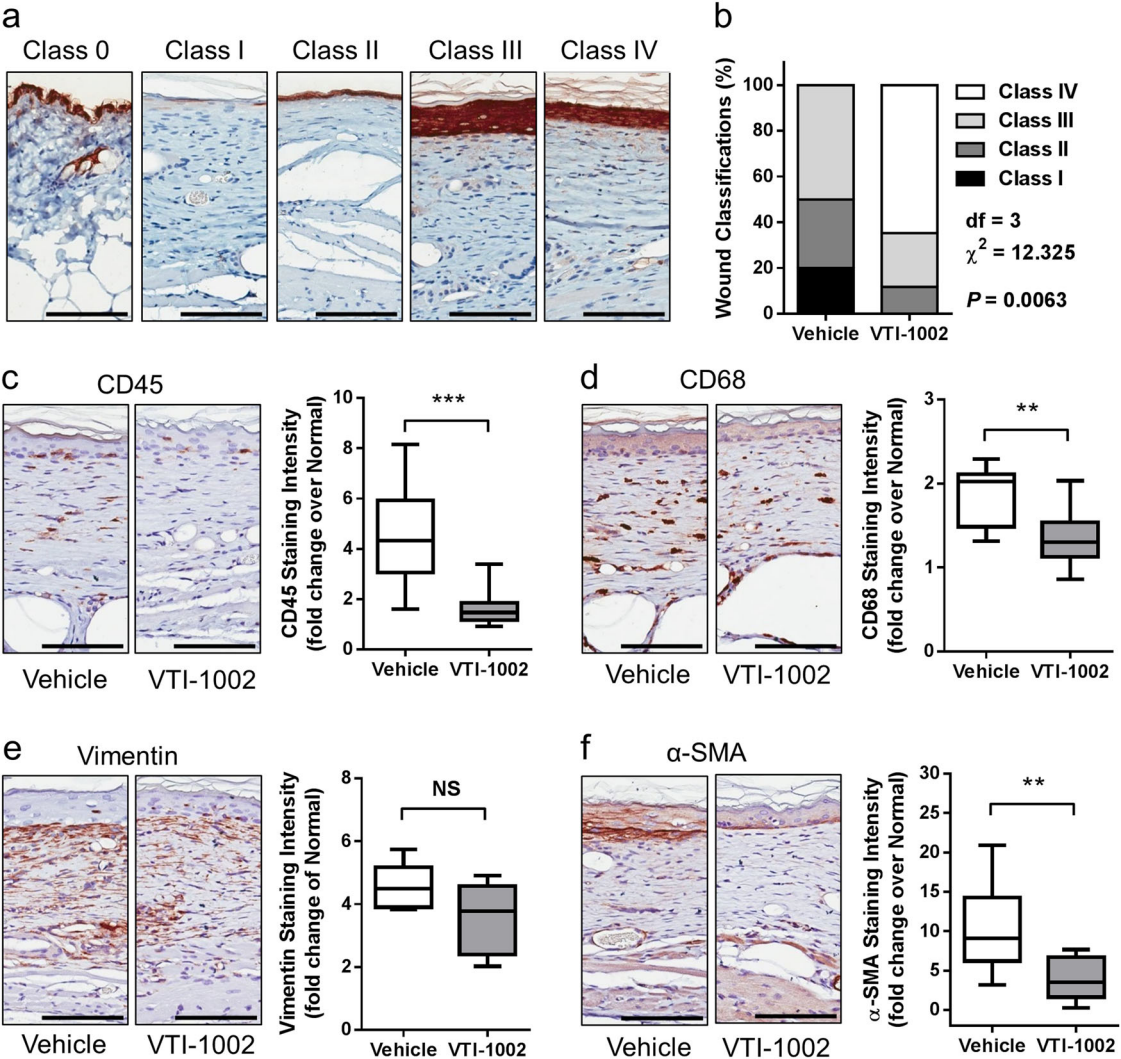
Topical application of VTI-1002 gel accelerates re-epithelialization and wound maturation in diabetic burn wounds. **a** Representative morphology of different wound classes (IHC keratin staining). **b** Prevalence of wound class in vehicle control group (*n* = 10) and VTI-1002 treatment group (*n* = 17). *P* = 0.0063 by *χ*^2^ test. Representative images of **c** CD45, **d** CD68, **e** vimentin, **f** α-SMA staining, and quantification of **c** CD45, **d** CD68, **e** Vimentin, **f** α-SMA staining intensity in the wounds at day 30 from vehicle control group and VTI-1002 treatment group (*n* ≥ 5 per group). Results are expressed as median and min/max value, NS = not significant, **P < 0.01, ***P < 0.001 by Student's *t* test. Scale bar = 100 µm

To further characterize macroscopic observations, histological cross-sections of wound tissues at day 30 were examined. During wound healing process, following fusion of the migrating keratinocyte layers, epidermal layer starts proliferating leading to hyper-proliferated epidermis, then epidermal hyper-proliferation decreases and epidermal thickness eventually returns to normal. Based on these well-characterized morphological changes during the wound healing process^34^, wound samples stained with keratin were categorized into five classes (Fig. 2a). As shown in Fig. 2b, all animals treated with VTI-1002 achieved full wound closure by the end of the experiment, and more than 50% of VTI-1002-treated wounds were categorized as class IV, an advanced stage of healing. Conversely, approximately half of the wounds in vehicle control remained open (class I) or newly closed (class II), suggesting that VTI-1002 gel treatment accelerated re-epithelialization and improves wound maturation (*P* = 0.0063 by *χ*^2^ test). In line with these findings, VTI-1002-treated wounds exhibited significantly lower levels of CD45 and CD68 staining, two major inflammatory cell markers, compared to vehicle-treated wounds (Fig. 2c, d). Immunostaining for vimentin and α-SMA, markers for activated fibroblast, further revealed a lower level of activated fibroblasts in VTI-1002-treated wounds versus vehicle-treated controls (Fig. 2e, f).

### Topical application of VTI-1002 gel reduces scar formation in diabetic burn wounds

**Fig. 3:**
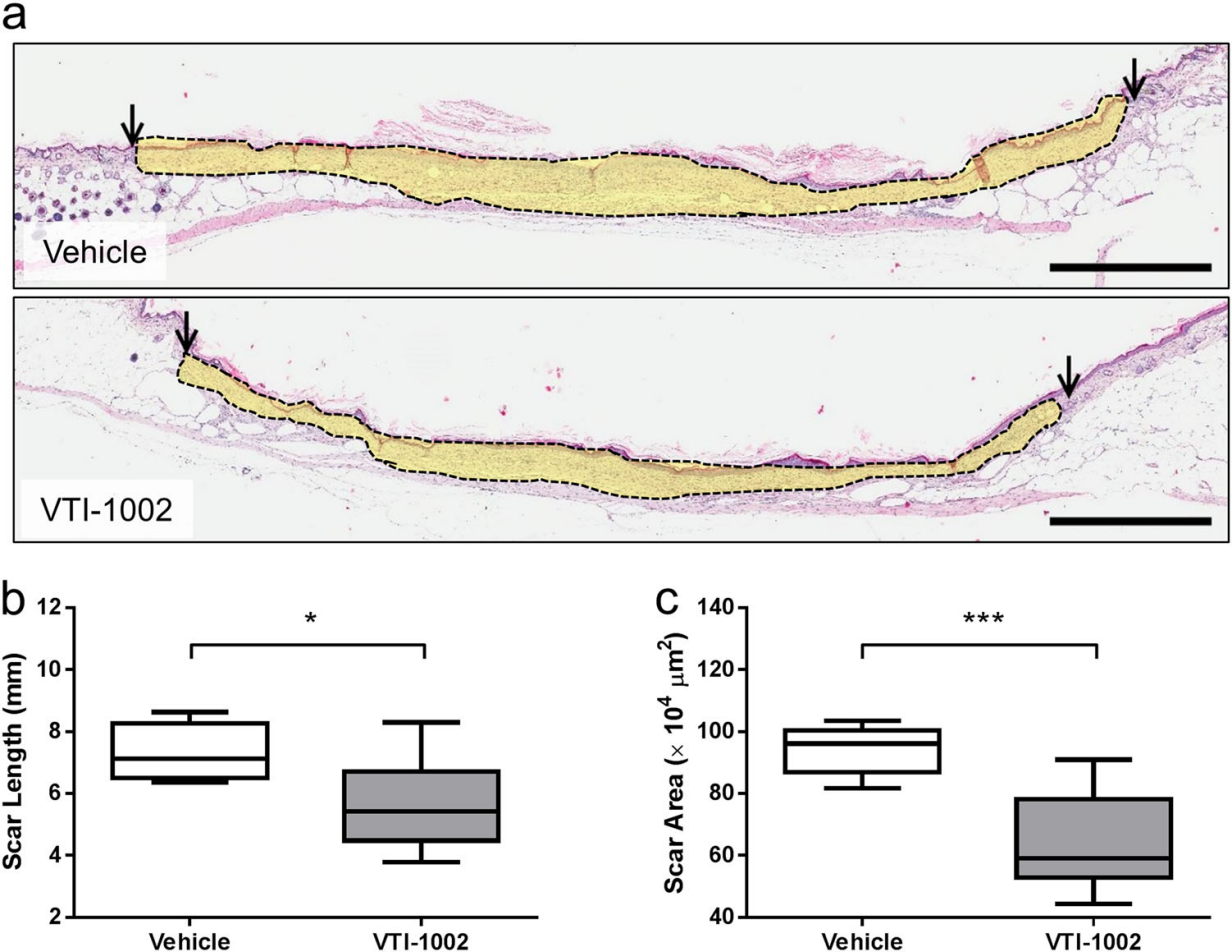
Topical application of VTI-1002 gel reduces scar formation in diabetic burn wounds. **a** Representative histological images of skin sections from day 30 wounds treated with vehicle and VTI-1002 gel. Area outlined by black line and light yellow color represented scar tissue. Scale bar = 1 mm. **b** Comparison of scar length at day 30 between vehicle control group and VTI-1002 treatment group (*n* ≥ 5 per group). **c** Comparison of scar area at day 30 between vehicle control group and VTI-1002 treatment group (*n* ≥ 5 per group). Results are expressed as median and min/max value, *P < 0.05, ***P < 0.001 by Student's *t* test

Scar formation is often an inevitable event during the healing of deep partial-thickness or full-thickness burn wounds. Permanent scars are detrimental to the function of tissue and often cause considerable painful, emotional, and social distress to patients^35^. At day 30, VTI-1002-treated animals exhibited reduced scarring compared to vehicle-treated controls (Fig. 3a). Scar length and area were also significantly reduced in the VTI-1002-treated mice (Fig. 3b, *P* = 0.0148; Fig. 3c, *P* **=** 0.0005).

### Topical application of VTI-1002 gel promotes tissue remodeling in diabetic burn wounds

**Fig. 4:**
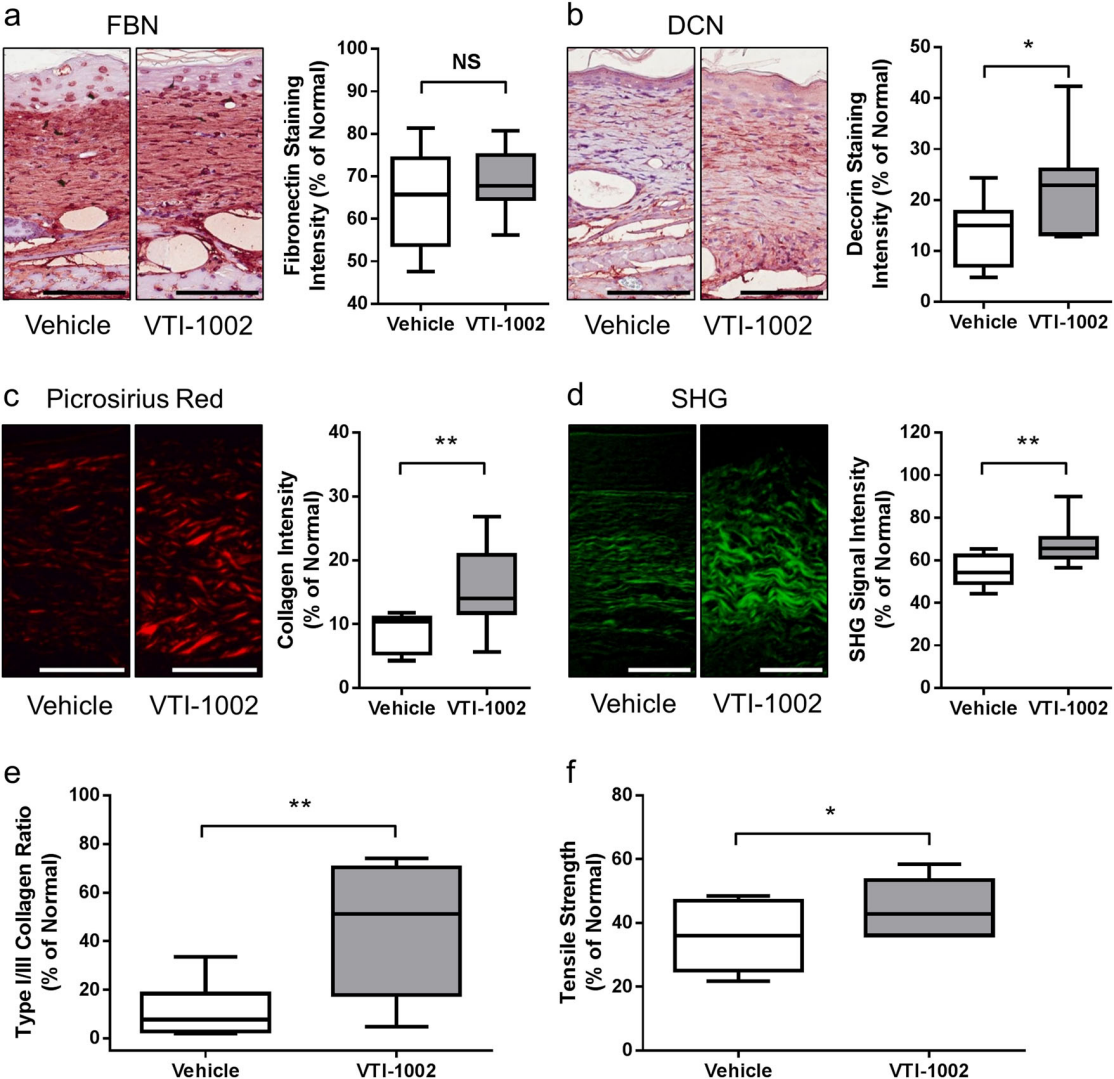
Topical application of VTI-1002 gel promotes tissue remodeling in diabetic burn wounds. Representative images of **a** FBN (fibronectin), **b** DCN (decorin) staining, and quantification of **a** FBN, **b** DCN staining intensity in the wounds at day 30 from vehicle control group and VTI-1002 treatment group (*n* ≥ 5 per group). Scale bar = 100 µm. **c** Representative images of Picrosirius Red staining and quantification of staining intensity in the wounds at day 30 from vehicle control group and VTI-1002 treatment group (*n* ≥ 5 per group). Scale bar = 100 µm. **d** Representative second-harmonic generation (SHG) imaging and quantification of SHG signal intensity in the wounds at day 30 from vehicle control group and VTI-1002 treatment group (*n* ≥ 5 per group). Scale bar = 50 µm. **e** Comparison of type I/III collagen ratio in the wounds at day 30 between vehicle control group and VTI-1002 treatment group (*n* ≥ 5 per group). **f** Comparison of post-wound skin tensile strength at day 30 between vehicle control group and VTI-1002 treatment group (*n* ≥ 5 per group). Results are expressed as median and min/max value, NS = not significant, *P < 0.05, **P < 0.01 by Student's *t* test

GzmB-mediated ECM cleavage has been proposed to be a key pathological event in several models of aging and impaired wound healing (reviewed in Ref.^11^). In the present study, there was no significant difference in FBN expression between VTI-1002-treated wounds compared to vehicle-treated controls at day 30 (Fig. 4a). However, immunostaining for DCN revealed a significantly higher level of DCN in VTI-1002-treated wounds versus vehicle-treated controls at day 30 (Fig. 4b). DCN plays an important role in skin collagen organization and tensile strength^36–38^. Collagen content in the wound at day 30 was examined using picrosirius red staining and SHG microscopy. Consistent with the DCN findings, VTI-1002-treated wounds exhibited a significantly greater level of collagen density and organization versus vehicle-treated controls (Fig. 4c, d). To further determine the maturity and quality of tissue remodeling, the ratio of collagens type I to type III in the wound was assessed using immunohistochemical staining. Topical application of VTI-1002 gel resulted in a significantly higher ratio of collagen type I versus type III, suggesting a more mature tissue modeling (Fig. 4e). In line with the above observations, VTI-1002 treatment also significantly increased the tensile strength of healed wounds, suggesting that VTI-1002-treated wounds would be less susceptible to re-injury (Fig. 4f).

### In vivo safety profile of 30-day topical VTI-1002 gel treatment

Animals that received daily topical VTI-1002 gel treatment did not exhibit any morbidity and mortality over the 30-day period. There were no signs of drug-induced adverse cutaneous reactions in either group at any time point (Table 2). Furthermore, VTI-1002 treatment group and vehicle control group showed no significant difference in their global health score (evaluated based on animal’s body weight, behavior, hydration, elimination, respiration, and pain. See detailed criteria in Supplementary Table 2) over the 30-day period (Supplementary Figure 2).

**Table 2:** VTI-1002 in vivo safety data

Group information	*n*	Route of admin	Treatment-related morbidity	Adverse cutaneous drug reaction	Mortality (no. of mice)		
					Exp. end-point	Humane end-point	Unexpected death
Vehicle	10	Topical	0	No observation of note	10	0	0
VTI-1002	18	Topical	0	No observation of note	18	0	0

## Discussion

The present study investigated the therapeutic effect of a novel, highly potent and specific, gel-formulated small-molecule GzmB inhibitor VTI-1002, on the healing of diabetic burn wounds. While previous serpin (SA3N)/knockout studies support GzmB as a therapeutic target, this is the first study to demonstrate feasibility using a topical GzmB inhibitor to accelerate wound healing and remodeling.

Wound healing is a well-orchestrated process that requires the integration of complex biological and molecular events in a spatial-specific and temporal-specific sequence^34^. In order for wound closure to ensue, keratinocytes migrate across the wound bed and, following fusion of the migrating keratinocyte layers, epidermal hyper-proliferation decreases and epidermal thickness gradually returns to normal. Mice from the VTI-1002 treatment group exhibited thinner hyper-proliferated epidermal layers and a more mature dermal extracellular matrix at day 30 when compared with vehicle controls, suggesting that wound healing in the VTI-1002 treatment group was more advanced than that in vehicle controls. Resolution of inflammation and reduction of activated fibroblast populations in a timely manner are also essential for successful wound healing^34^. VTI-1002-treated wounds exhibited reduced inflammation and fibroblasts compared to the vehicle-treated group, indicating that VTI-1002 treatment accelerates diabetic wound maturation.

The remodeling process in chronic wounds is known to be disrupted by a pathologic cycle of inflammation and protease release leading to the degradation of ECM proteins, further tissue damage, and exacerbation of inflammation^39^. It is believed that loss of essential ECM proteins in chronic wounds is mainly due to increased proteolytic activity as opposed to reduced synthesis^40,41^. MMPs have long been proposed as key therapeutic targets for chronic diabetic wound treatment^6^. However, it is now clear that MMP activity is essential for wound healing. In fact, broad inhibition of MMPs promotes inflammation by suppressing MMP-mediated chemokine regulation^42,43^. Furthermore, approximately half of the known MMPs are now proposed to be drug anti-targets, that is, their activities should be promoted, not inhibited in wound healing therapies^43^. GzmB contributes to the pathogenesis of chronic wounds by cleaving ECM proteins essential for healing to ensue^9,10^. In contrast to MMPs and other resident extracellular proteases found in wound fluid, GzmB is not normally found in high levels in the extracellular milieu, and is one of the only extracellular serine proteases to which no endogenous extracellular inhibitor has been identified in humans^11^. As extracellular proteolytic activity is a tightly regulated network and extracellular GzmB is found in minimal to absent levels in healthy individuals^24^, we hypothesize that pharmacologic extracellular GzmB inhibition promotes wound closure and tissue remodeling.

One of the major clinical outcomes of deep partial-thickness to full-thickness thermal burn wounds is scarring, which is estimated to occur between 32% to over 70% of burn injuries^44^. Permanent scars present a huge burden to the patient both physically and emotionally due to pain, pruritus, contractures, loss of function, and disfigurement^35^. DCN, a small, leucine-rich proteoglycan, is the most abundant proteoglycan in the skin and plays an important role in preventing scar formation^36^. DCN expression is suppressed during scarring and increases when hypertrophic scarring is resolved^23^. Furthermore, reduced DCN is observed in post-burn hypertrophic scarring and loss of DCN has been proposed to contribute to hypertrophic scarring^45,46^. As further support, scarring and fibrosis are exacerbated in DCN knockout mice^47,48^. Excessive GzmB-mediated DCN degradation during wound healing negatively impacts collagen deposition and remodeling^9^. In the present study, wounds from VTI-1002 treatment group exhibited greater levels of DCN compared with those from vehicle controls implying that GzmB inhibition prevents the loss of DCN in post-burn collagen remodeling. In line with previous findings, increased DCN was also associated with significantly greater collagen density and organization in VTI-1002-treated wounds compared with vehicle controls. Interestingly, DCN knockout mice exhibit reduced collagen spacing, organization, and tensile strength^37^. Of particular interest in our study, topical VTI-1002 significantly improved post-burn skin tensile strength in concordance with the collagen/DCN histological data. Given that chronic wounds rarely heal to original tensile strength thereby predisposing wounds to further injury, it is exciting to speculate that inhibition of GzmB-mediated DCN cleavage could promote increased tensile strength and reduce the incidence of injury re-occurrence.

One of the greatest advantages of a topical therapy for burn and/or chronic wound treatment is that it is non-invasive and easy to apply versus systemic approaches. Topically applied VTI-1002 was retained in skin for at least 24 h, and the systemic absorption of VTI-1002 (as measured by plasma concentration) was minimally detectable. Furthermore, despite the repeated administration of VTI-1002 gel to the skin, test animals did not exhibit any adverse events and/or signs of discomfort during the application process.

Similar to all murine disease models, there are some limitations associated with the diabetic (*db/db*) mouse model of burn wound healing. The *db/db* mouse model is a monogenic model of type II diabetes and demonstrates a severe obesity profile, whereas type II diabetes in humans is a polygenic disease^49^. In addition, unlike diabetic patients, the level of hyperglycemia in *db/db* mice does not correlate with the severity of impaired wound healing^50^. Nevertheless, the *db/db* mouse model of type II diabetes is one of the most commonly used model of impaired wound healing. In our study, it provides valuable insights into the therapeutic efficacy of a GzmB inhibitor on diabetic burn wounds, which demonstrates proof of concept and serves as a stepping stone towards further clinical development. Although additional studies are required to more thoroughly investigate the toxicology, pharmacokinetics, and efficacy of VTI-1002 gel before the commencement of clinical trials, the present results provide a foundation to support further development toward the clinic.

## Electronic supplementary material

Supplemental Material
