# Supplementary material for: Topical small molecule granzyme B inhibitor improves remodeling in a murine model of impaired burn wound healing
Source: Exp Mol Med. 2018 May 30;50(5):68. doi: 10.1038/s12276-018-0095-0 (PMC5976625; doi:10.1038/s12276-018-0095-0)
Supplement: Supplementary file 1 — Supplemental Material [file 12276_2018_95_MOESM1_ESM.pdf]

Supplementary Information

Topical Small-molecule Granzyme B Inhibitor Improves Remodeling in A Murine Model of Impaired Burn Wound Healing

Running Title: Novel Therapy for Burn Injury and Impaired Healing

Authors:

Yue Shen[1,2,3,6], Matthew R. Zeglinski[1,2,3], Christopher T. Turner[1,2,3], Sheetal A. Raithatha[1,6], Zhengguo Wu[4,5], Valerio Russo[1,2,3], Cameron Oram[1,2,3], Sho Hiroyasu[2,3], Layla Nabai[2,3], Hongyan Zhao[1,2,3], Tatjana Bozin[1], Kathryn Westendorf[1,6], Irina Kopko[7], Rachel Huang[7], Steve Arns[7], Jason Tan[7], Haishan Zeng[4,5], Anthony Boey[7], Richard Liggins[7], James Jaquith[7], Dale R. Cameron[6], Anthony Papp[3], David J. Granville[1,2,3,6*]

Affiliations:

[1]Centre for Heart Lung Innovation, St. Paul's Hospital, University of British Columbia, Vancouver, BC, Canada.

[2]International Collaboration On Repair Discoveries (ICORD), Vancouver Coastal Health Research Institute and Department of Pathology and Laboratory Medicine, University of British Columbia, Vancouver, BC, Canada.

[3]BC Professional Firefighters' Burn and Wound Healing Group, Vancouver, BC, Canada.

[4]Imaging Unit, Integrative Oncology Department, BC Cancer Agency Research Centre, Vancouver, BC, Canada.

[5]Photomedicine Institute, Department of Dermatology and Skin Science, University of British Columbia & Vancouver Coastal Health Research Institute, Vancouver, BC, Canada;

[6]viDA Therapeutics, Inc. Vancouver, BC, Canada

[7]Centre for Drug Research and Development, Vancouver, BC, Canada

*To whom correspondence should be addressed:
David J Granville, ICORD Centre, Rm 4470, 818 West 10th Ave.
Vancouver, BC, V5Z 1M9, CANADA. Tel: +1-604-806-9267; Fax: +1-604-806-8351; Email. dgranville@icord.org

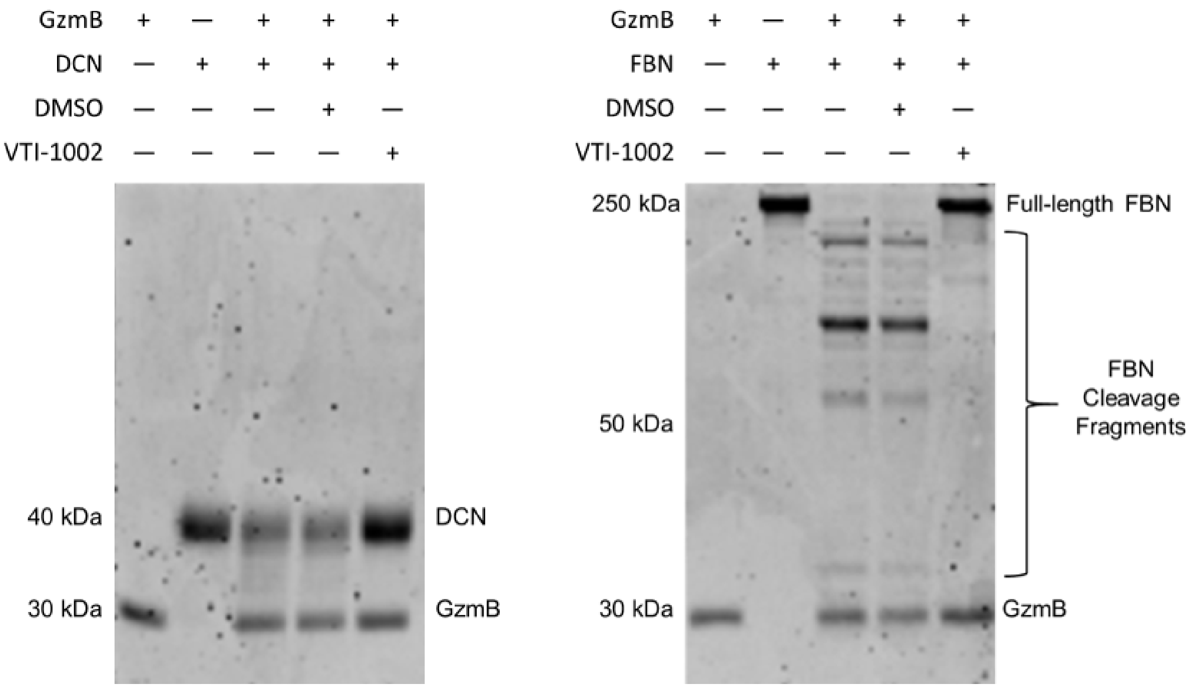

Supplementary Figure 1. *In vitro* Characterization of VTI-1002 activity. VTI-1002 abolishes GzmB-mediated ECM cleavage. (left) GzmB-mediated DCN cleavage assay; (right) GzmB-mediated FBN cleavage assay.

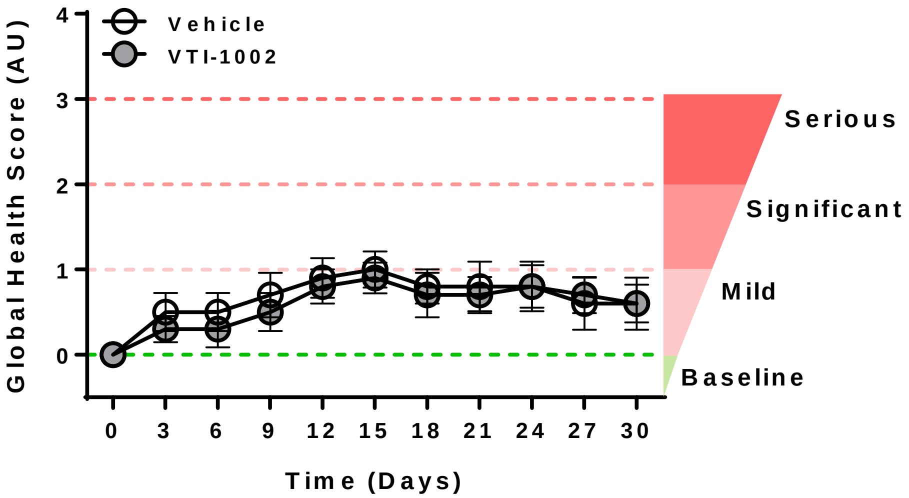

Supplementary Figure 2. Topical VTI-1002 gel treatment does not affect global health. Comparison of global health score over the 30-day healing period between vehicle control group and VTI-1002 treatment group (n = 10 per group). Global health score is the sum of scores of each health observation category (**Supp. Table 2**). Score 0 indicates the baseline health (normal); Score 1 indicates mild health impact; Score 2 indicates significant health impact; Score 3 indicates serious health impact (humane endpoint).

Supplementary Table 1: Plasma level of VTI-1002 after topical application

Time Point	4 hrs			6 hrs			24 hrs		
Sample #	1	2	3	4	5	6	7	8	9
Plasma (ng/mL)	1.6	BLOQ	BLOQ	7.6	7.4	BLOQ	ND	BLOQ	3.4

BLOQ: Below the limit of quantitation (1 ng/mL in plasma)

ND: Not detected.

Supplementary Table 2: Global Health Score Reference Chart

Health Observation Categories	Health Score			
	0<br><br>(Baseline health)	1<br><br>(Mild)	2<br><br>(Significant)	3<br><br>(Serious, Humane endpoint)
Body weight	↓weight by 0-4%	↓weight by 5- 9%	↓weight by 10-14%	↓weight over 15%
Wound site	Occasional scratching	Redness or scaling	Skin inflammation, drug-induced rash	Severe skin inflammation, ulceration
Behavior	Intermittent stereotypic behaviour during cage changing; BAR (Bright, Alert, Responsive)	Slightly slow moving; still interested in environment; QAR (Quiet, Alert, Responsive)	Less interested in the environment, interacts less with cage mates, disregards observer; when nudged, reluctantly moves away. Occasional abnormal gait	Isolated from cage mates, minimally active; does not readily move when cage disturbed. When nudged reluctantly moves. Limping or “tip-toed” gait.
Dehydration	N/A	Mildly sunken eyes (appear >75% open).	Skin tent > 2 seconds (decrease in skin elasticity). Sunken eyes (appear half closed).	Skin tent > 5 seconds. Completely closed or severely sunken eyes; tail feels square. Cool to touch.
Elimination<br><br>(Feces, Urine)	Soft but formed feces, hydration normal	Daily paste-like feces, hydration normal	Daily watery diarrhea, occasionally formed, soft feces, increased urine output. Small area of fecal anus staining, urine scalding. Hydration normal	Watery diarrhea or increased urination resulting in dehydration; larger area of fecal staining, urine scalding, fecal smearing on walls
Respiration	Occasional increased rate	Subtle change in rate or effort	Obvious change in rate or effort	Reduced rate, irregular rhythm;

	after activity; normal rhythm and effort	with activity; normal at rest; regular rhythm	with activity and at rest; occasional irregular rhythm; small amount of nasal discharge, sneezing	requires effort to breathe (abdominal effort); noisy breathing; nasal discharge affecting breathing
Pain	None	N/A	Facial grimace: narrow or closed eyes, bulge on top of nose, cheek bulge, ears back or flat, whiskers pointing back or "standing out on end"<br>Other: muscle twitching or flinching, staggering, back stretch, abdominal writhing or pressing	Persistent signs of pain that interfere with normal functions or cannot be alleviated
